# Mass spectrometry-based analysis of gut microbial metabolites of aromatic amino acids

**DOI:** 10.1016/j.csbj.2023.09.032

**Published:** 2023-09-26

**Authors:** Narumol Jariyasopit, Sakda Khoomrung

**Affiliations:** a Siriraj Center of Research Excellence in Metabolomics and Systems Biology (SiCORE-MSB), Faculty of Medicine Siriraj Hospital Mahidol University, Bangkok 10700, Thailand; b Siriraj Metabolomics and Phenomics Center, Faculty of Medicine Siriraj Hospital Mahidol University, Bangkok 10700, Thailand; c Department of Biochemistry, Faculty of Medicine Siriraj Hospital Mahidol University, Bangkok 10700, Thailand

**Keywords:** Gut microbial metabolite, Metabolomics, Aromatic amino acid, Mass spectrometry, Quantitative analysis

## Abstract

Small molecules derived from gut microbiota have been increasingly investigated to better understand the functional roles of the human gut microbiome. Microbial metabolites of aromatic amino acids (AAA) have been linked to many diseases, such as metabolic disorders, chronic kidney diseases, inflammatory bowel disease, diabetes, and cancer. Important microbial AAA metabolites are often discovered via global metabolite profiling of biological specimens collected from humans or animal models. Subsequent metabolite identity confirmation and absolute quantification using targeted analysis enable comparisons across different studies, which can lead to the establishment of threshold concentrations of potential metabolite biomarkers. Owing to their excellent selectivity and sensitivity, hyphenated mass spectrometry (MS) techniques are often employed to identify and quantify AAA metabolites in various biological matrices. Here, we summarize the developments over the past five years in MS-based methodology for analyzing gut microbiota-derived AAA. Sample preparation, method validation, analytical performance, and statistical methods for correlation analysis are discussed, along with future perspectives.

## Graphical Abstract

**Figure:**
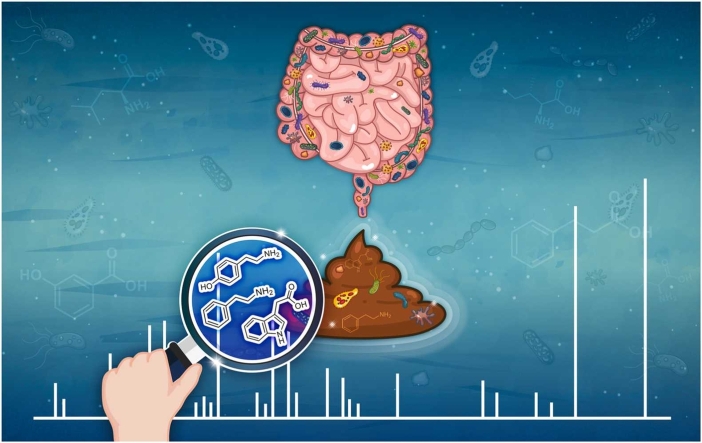

## 1 Introduction

Gut microbiota, the predominant microbial community in the human body, is highly diverse and comprises up to 1150 species [1]. Gut microbiota play important roles in symbiotic conditions and disease pathogenesis [2], [3]. Investigating gut microbial metabolites (molecules < 1500 Da) [4] can provide deep insights into their connections to human health and diseases. Typically, these metabolites are synthesized by gut microbes themselves or from dietary molecules, or co-metabolized by the host and gut microbes [1].

Among the gut microbial metabolites, amino acids (AA) and their metabolites were found to substantially affect the gut and distant organs, and contribute to disease pathogenesis [5], [6], [7], [8], [9], [10], [11]. Aromatic amino acids (AAA), including phenylalanine (Phe), tryptophan (Trp), and tyrosine (Tyr), are essential nutrients for humans and are naturally produced by plants and microorganisms [12], [13], [14]. Humans can obtain Phe and Trp from dietary proteins while Tyr can be produced endogenously from Phe via phenylalanine hydroxylase in the liver [15], [16]. After entering the body, AAA are metabolized by host and gut microbiota [13], [16], [17]. A list of gut microbial AAA metabolites, bacterial species, and enzymes involved in Phe, Tyr, and Trp metabolism has been summarized [16].

The interplay between host and gut microbiota with regard to AAA has been linked to the pathogenesis of metabolic disorders [8], type 2 diabetes (T2D) [11], liver diseases [7], chronic kidney diseases [6], [10], and neurological diseases [5], [9]. The gut microbial metabolism in AAA and its association with disease have recently been reviewed by [16].

Functional roles of the gut microbiota have been investigated through gut microbial metabolomics using technologies that have improved over the past decades. Two main analytical platforms, nuclear magnetic resonance (NMR) spectroscopy and mass spectrometry (MS), are often recommended for metabolomic analyses [18]. Because of minimal sample pretreatment requirements, NMR facilitates high-throughput metabolomic analysis, but at the expense of sensitivity. Nonetheless, NMR is suitable for both ionizable and non-ionizable metabolites and can provide definite structures for the concerned metabolites. Alternatively, the compatibility of MS with chromatography systems, together with sample pretreatment, considerably enhances the analytical performance via multi-dimensional separation channels. Typically, the detection limits of MS are approximately 1–2 orders of magnitude lower than those of NMR [19]. This high sensitivity makes it suitable for both untargeted and targeted metabolomic analyses.

**Fig. 1:**
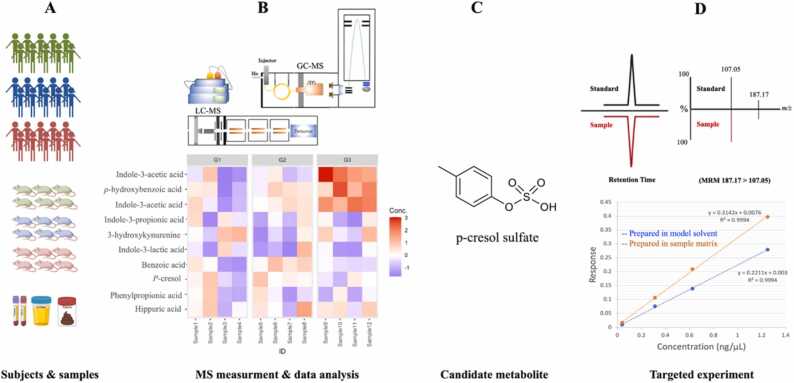
Overview of workflow for discovery and validation of gut microbial metabolites in clinical research. A: study design and sample collection, B: sample preparation and metabolite profiling via untargeted or semi-targeted analysis using MS-based methods followed by substantial statistical analysis, C: proposed candidate metabolites, D: Targeted analysis to confirm metabolite identity and determine their absolute concentrations.

Generally, for a given cohort study (human or animal), MS-based untargeted analysis of biological specimens is applied to preliminarily search for potential biomarkers (Fig. 1). Though the potential biomarker identified through the untargeted analysis can be limited by the choice of analytical techniques and availability of mass spectral libraries, it offers overall metabolite profiles without prior knowledge of the metabolites involved in the research questions. The candidate metabolites identified at this stage require further confirmation and quantification with reference standards using a targeted approach [20]. The validity of biomarkers should be confirmed using various types of biological validation, such as animal models, bacterial cultures, and larger cohorts [21], [22]. Absolute quantitative analysis of validated biomarkers allows for comparison across different studies, which eventually leads to the establishment of threshold concentrations and facilitates the translation of potential metabolite biomarkers into clinical practice. This underscores the importance of quantitative metabolomics in bridging scientific outcomes with clinical applications.

However, major challenges in quantitative metabolomics include the availability of authentic reference standards and lack of standardized methods [23]. MS-based quantitative methods are typically optimized according to sample matrices and available MS platforms. Therefore, different methods can measure similar compounds, posing difficulties in the standardization of metabolomic protocols across different laboratories. Nonetheless, the development of a reliable quantitative MS-based method usually adheres to analytical guidelines that include a series of validation tests: method recovery, inter- and intra-day variations, linearity, specificity, matrix effects, analyte stability, carryover, and determination of the limit of detection and quantification [20].

In response to the increasing interests in gut microbial metabolites, analytical methods for analyzing short-chain fatty acid, bile acids, and methylamines have been widely developed and summarized [24], [25]. In contrast, MS-based metabolomic methods for qualitative and quantitative analyses of gut microbial AAA metabolites have not been reviewed. Thus, the scope of this review is to summarize the developments over the past five years in MS-based methodologies for profiling and quantifying gut microbial metabolites of AAA. We discuss the general guidelines of sample preparation, method validation, and analytical performance for quantitative analysis. Finally, we summarize the statistical methods often used for metabolome-microbiome correlation analyses and offer future perspectives.

## 2 Metabolite profiling and targeted analysis

Table 1 summarizes gut microbial metabolites of AAA identified in various types of biological specimens collected from rats, mice, and humans using gas chromatography – MS (GC-MS) and liquid chromatography – MS (LC-MS).

**Table 1:** Summary of gut microbial AAA metabolite analytical methods. ESI modes of analysis are provided in parenthesis. Abbreviations: IT, Ion-trap; TOF, Time-of-flight; Q, quadrupole; SQ, single quadrupole; TRAP, orbitrap; QTRAP, quadrupole and orbitrap.

Tyrosine metabolites	Tryptophan metabolites	Phenylalanine metabolites	Model	Matrix quantified	Analytical Platform	Column Mobile phases	Derivatizing agent	Ref
ferulic acid (-)4-hydroxycinnamic acid (-) 4-hydroxyphenylacetic acid (-) 3-hydroxybenzoic acid (-) 4-hydroxyphenylpropionic acid (-)	indole-3-acetic acid (+)indole-3-aldehyde (-)indole-3-acrylic acid (+) indole-3-propionic acid (+)	benzoic acid (-)hippuric acid (-)phenylpyruvic acid (-)phenyllactic acid (-)phenylacrylic acid (-)phenylpropionic acid (-)phenylacetylglutamine phenylacetic acid (-)	Rat (T2D)	Serum, urine, feces	LC-MSMS (TQ)	Phenyl-Hexyl column (150 × 2.1 mm, 1.7 µm) Solvent A: water 2 mM ammonium formate Solvent B: methanol		Zou et al. 2021
	3-hydroxykynurenine (+) serotonin (+) 5-hydroxytryptophan (+) 5-hydroxyindoleacetic (+) acid tryptamine (+) indole-3-acetic acid (+) indoxyl sulfate (+)indole-3-acetamide (+) indole-3-lactic acid (+) indole-3-carboxaldehyde (+) indole-3-acetic acid (+) tryptophol (+)		Mouse (healthy)	Serum, urine, intestinal contents, liver	UPLC-MS/MS (HRMS, QTRAP)	XB – C18 (150 mm × 2.10 mm, 1.7 µm, 100 Å) Solvent A: 0.5% (v/v) formic acid in water Solvent B: methanol with 0.5% (v/v) formic acid		Lefevre et al. 2019
p-cresol (-)p-cresyl sulfate (-)	indole (+)indoxyl sulfate (-)	*p*-cresol (-)*p*-cresyl sulfate (-)	Rat (Crohn's disease)	Serum, Urine	HPLC-MS/MS (TQ)	Phenyl-Hexyl column (50 × 2.1 mm, 1.7 µm) Solvent A: water with 5 mmol/L ammonium formate and 0.05% formic acid Solvent B: methanol		Zeng et al. 2017
p-hydroxybenzoic acid (-)p-hydroxyphenylacetic acid (-)	indole-3-acetic acid (-)indole-3-propionic acid (-)5-hydroxyindoleacetic acid (-)	benzoic acid (-)phenylacetic acid (-)phenylpropionic acid (-)	Rat (high-fat diet-induced obesity)	Serum	HPLC-MS/MS (TQ)	BDS Hypersil C18 column (50 ×2.1 mm, 2.4 µm) Solvent A: 0.05% formic acid in water Solvent B: methanol		Zeng et al. 2019
p-hydroxyphenylpropionic acid (+)p-hydroxybenzoic acid (+)p-hydroxyphenyllactic acid (+) p-hydroxyphenylacetic acid (+) p-ethylphenol (+) p-coumaric acid (+) phenol (+)tyramine (+)p-cresol glucunoride (+) p-cresol (+)p-cresol sulfate (+)		*p*-cresol (+)*p*-cresol sulfate (-)	Mouse (high tyrosine diet)	Plasma, urine	UPLC-MSMS (TQ)	BEH C8 (1 mm × 100 mm, 1.7 µm) Solvent A: 0.1% (v/v) formic acid in water Solvent B: 0.1% (v/v) formic acid in acetonitrile: isopropanol (1:1 v/v)	Dansyl chloride	Letertre et al. 2021
	Quantitative analysis indole-3-acetic acid (+) methyl indole-3-acetic acid (+)methyl indole-3-propionic acid (+) Semi-quantitative analysis 5-hydroxyindoleacetic acid (+)5-hydroxy-L-tryptophan (+)indole-3-acetamide (+)tryptophol, indole-3-ethanol (+)indole-3-propionic acid (+)serotonin (+)tryptamine (+)		Human (pregnant women)	Urine	UHPLC-MS/MS (TQ)	C18 CSH (2.1 mm i.d. 100 mm, 1.7 µm) Solvent A: water Solvent B: acetonitrile with 0.1% formic acid		Pavlova et al. 2017
ferulic acid (-)4-hydroxycinnamic acid (-)3-hydroxybenzoic acid (-)4-hydroxybenzoic acid (-)4-hydroxyphenylacetate (-)p-hydroxyphenylpropionic acid (-)		phenylacetic acid (-)phenylpropionic acid (-)hippurate (-)	Human (healthy subjects after the consumption of orange juice)	Urine	UHPLC-MS (TRAP)	C18 (150 × 4.6 mm i.d., 5 µm, 100 Å) 0.1% acidic methanol in 0.1% aqueous formic acid		Ordonez et al. 2018
	indoxyl sulfate	*p*-cresol sulphatehippuric acidphenylacetylglycine	Human (end state renal disease)	Serum	UHPLC-MS/MS (TQ)	Discovery HS F5–3 column (150 × 2.1 mm, 3 µm) Mobile phases: not provided.		Wang et al. 2020
	indoxyl sulfate (-)	phenylacetylglutamine (-) hippuric acid (-)	Human (postmenopause female subjects taking a green tea polyphenol supplement	Feces, urine	UPLC-MS/MS (QTOF)	BEH C18 column Fecal: Solvent A: 0.05% (v/v) aqueous acetic acid with 5 mM ammonium acetate Solvent B: 95% (v/v) aqueous acetonitrile with 0.05% acetic acid and 5 mM ammonium acetate Urine: Solvent A: 0.1% formic acid Solvent B: acetonitrile with 0.1% formic acid		Zhou et al. 2019
4-hydroxyphenylpyruvic acid (-)4-hydroxyphenyllactic acid (-)4-hydroxyphenylacrylic acid (-) 4-hydroxyphenylpropionic acid (-)4-hydroxyphenylacetic acid (-)	indolepyruvic acid (-)indole-3-acetic acid (-)indole-3-acrylic acid (-)indole-3-propionic acid (-)indole-3-lactic acid (-)	phenylpyruvic acid (-)phenyllactic acid (-)phenylacrylic acid (-)phenylpropionic acid (-)phenylacetatic acid (-)	Mouse, Bacterial culture	Serum, cecal contents	LC-MSMS (TQ)	poroshell 120 C18 column (4.6 × 50 mm) Solvent A: 0.1% formic acid (v/v) in water Solvent B: acetonitrile		Dodd et al. 2017
	indole-3-propionic acid (+)indole-3-acetic acid (+)indole-3-carboxaldehyde (indole-3-aldehyde) (+)indole-3-lactic acid (+)indole-3-acetamide (+)indoxyl sulfate (+)tryptamine (+)serotonin (+)5-hydroxytryptophan (+)		Human (healthy)	Plasma (adults), urine (children)	LC-MS/MS (TQ)	ACQUITY HSST3 (1.8 µm, 2.1 × 150 mm) Solvent A: 0.1% formic acid in water Solvent B: acetonitrile with 0.1% formic acid		Anesi 2019
ferrulic acid4-hydroxycinnamic acid4-hydroxybenzoic acid3-hydroxybenzoic acid4-hydroxyphenylacetic acid3-(p-hydroxyphenyl)propionate, p-hydroxyphenylpropionic acid		phenylacetic acidhippuric acid	Human (healthy subjects after the consumption of orange juice)	Urine	GC-MS (IT)	ZB-5MS (30 m × 0.25 mm × 0.25 µm film thickness)	MSTFA	Ordonez et al. 2018
4-hydroxycinnamic acidp-hydroxybenzoic acidp-hydroxyphenylacetic acidp-hydroxyphenyllactic acidp-cresolphenol4-Hydroxyphenylpyruvic acid	indole-3-acetic acidindole-3-propionateindole-3-acrylic acid5-hydroxytryptophanserotonintryptamineindole	phenyllactic acidphenylacetic acidhippuric acid*p*-cresolphenylethylaminephenylpyruvic acid	Human (healthy), Bacterial cell	Serum, urine, feces, Escherichia coli cell samples	GC-MS (TOF)	DB-5 MS capillary column (30 m × 0.25 mm i.d., 0.25 µm film thickness)	Alkyl chloroformate	Zhao et al. 2017
p-hydroxyphenylacetic acidp-hydroxyphenyllactic acidp-hydroxyphenylpropionic acid4-hydroxybenzoic acid		benzoic acidphenylpropionic acidphenyllactic acid	Human (acute critical ill patients with nosocomial pneumonia, chronically critically ill patients)	Serum, feces	GC-MS (Q)	TR-5MS capillary column (30 m × 0.25 mm, 0.25 µm film thickness)	BSTFA	Chernevskaya et al. 2020
p-hydroxyphenylacetic acidp-hydroxyphenylpropionic acid4-hydroxybenzoic acid4-hydroxyphenyllactic acid		benzoic acidphenylpropionic acidphenyllactic acid	Human (healthy)	Serum	GC-MS (Q)	TR-5 ms quartz capillary column (30 m × 0.25 mm, 0.25 µm film thickness)	BSTFA	Pautova et al. 2018
	indole-3-acetic acidindole-3-propionic acidIndole-3-lactic acid5-hydroxyindole-3-acetic acid		Human (central nervous system disease)	Serum, cerebrospinal fluid	GC-MS (Q)	TR-5MS capillary column (30 m × 0.25 mm, 0.25 µm film thickness)	BSTFA, MTBSTFA	Pautova et al. 2020
4-hydroxyphenylacetic acid	indole-3-acetic acidindole-3-lactic acidindole-3-propionic acid	hippuric acidbenzoic acid phenylethylamine	Rat (Rhubarb-treated)	Feces	GC-MS (TOF)	DB-5 ms capillary column (30 m, 0.25 mm i.d. 0.25 mm 0.25 µm film thickness)	BSTFA	Yin et al. 2017

### 2.1 GC-MS

#### 2.1.1 Sample preparation

##### 2.1.1.1 Serum

The volume of serum used range between 40 and 200 µL. Serum preparation often begins with protein precipitation using either an organic solvent (e.g., methanol (MeOH)) or an acidified solution (e.g., H_2_SO_4_) after diluting the serum with water [26], [27], [28]. Protein precipitation is a sample pretreatment used to remove proteins without eliminating the metabolites of interest. This process decreases the interference from unwanted species and extends the lifetime of the column and mass detector. However, protein precipitation can impair the reproducibility or accuracy of analytical methods if the metabolites of interest have high affinities for proteins [29]. Subsequent to protein precipitation, simple extraction with MeOH or diethyl ether (DE) using liquid-liquid extraction or centrifugation is often recommended. However, Pautova et al. extracted indolic acids, which are gut microbial metabolites of Trp, from serum and cerebrospinal fluid without protein precipitation using microextraction by packed sorbent (MEPS) [9]. MEPS is a solid-phase extraction (SPE) technique that is used for small-volume extraction and purification. Unlike SPE columns, MEPS is reusable and can be fully automated followed by GC-MS or liquid chromatography (LC)-MS. This technique reduces the serum matrix effect on indolic acid analysis. However, it adds additional steps to the conditions, including washing and drying of the MEPS column, which requires additional development and validation.

##### 2.1.1.2 Urine

Urinary AAA metabolites can be extracted by SPE using reversed-phase SPE cartridges after acidification of urine with phosphoric acid [30]. Another study lyophilized urine samples and reconstituted them in 1 M sodium hydroxide (NaOH) and MeOH or ethanol before derivatization [26]. The same study also reported that lyophilization of urine samples could enhance the intensities of target metabolites across the chromatogram and increase the number of identified metabolites.

##### 2.1.1.3 Feces

Owing to the heterogeneity of fecal samples, the sample pretreatment protocol is important, and the amount of sample must be optimized to avoid column overloading and detector saturation. Zhao et al. homogenized 10 mg of freeze-dried human fecal samples with NaOH solution before centrifugation [26]. After collecting the supernatant, the sample was re-extracted with cold MeOH and the two extracts were combined. Yin et al. [31] performed a series of experiments to optimize the amount of rat feces and extraction solvent combinations. Multivariate analysis of fecal metabolite profiles suggested that a higher proportion of MeOH in the MeOH/chloroform extraction mixture was better for gut microbial metabolites of AAA, but a lower proportion of MeOH was more suitable for metabolites in the tricarboxylic cycle. However, a combination of MeOH, chloroform, and water (225:75:300, v/v/v) was chosen in their study because it is suitable for a wide range of metabolites; however, this could compromise the sensitivity of this method.

##### 2.1.1.4 Bacterial cultures

Following untargeted or metabolite profiling analyses, key AAA metabolites identified in human samples can be validated using bacterial cultures to determine their roles in metabolic pathways. To accurately measure intracellular metabolites from bacteria or other microorganisms, samples must be quenched to prevent metabolite turnover and minimize leakage of intracellular metabolites [32], [33]. The most common quenching methods for bacterial metabolomics are cold MeOH solution, cold glycerol-saline solution, and liquid nitrogen [34]. Most of previous quenching methods were optimized for untargeted metabolomic analysis, not specifically for the analysis of AAA metabolites. However, a fast-quenching method using liquid nitrogen in combination with a filter-based sampling was shown to be suitable for intracellular amino acid quantification in bacteria [35]. After collecting bacterial cells and removing the culture medium, Zhao et al. quenched the bacterial cells by washing with phosphate-buffered saline (PBS) before reconstituting them in PBS [26]. Cell lysates were homogenized with water and extracted with cold MeOH via centrifugation. Although this high-throughput GC-MS based method was able to measure AAA metabolites in serum, urine, and feces, the metabolites were not detected in the bacterial cells used in the study.

Typically, polar and nonvolatile metabolites, such as AA, free fatty acids, and AAA microbial metabolites, require chemical derivatization to modify their structures for GC-MS analysis. Silylation-based derivatization reagents such as *N,O*-Bis(trimethylsilyl)trifluoroacetamide (BSTFA), *N*-Methyl-*N*-(trimethylsilyl)trifluoroacetamide (MSTFA), and *N*-Methyl-*N*-tert-butyldimethylsilyltrifluoroacetamide (MTBSTFA) are often used [36]. The silylation reaction replaces the labile hydrogen (i.e., -COOH, -OH, -NH, NH_2_, and-SH) with a trimethylsilyl (TMS) moiety, producing less polar and more volatile TMS derivatives. Based on the previous literature and our experience, the important drawbacks of silylation using TMS reagents are as follows: I) TMS reagents and derivatives are sensitive to moisture, II) TMS derivatives are stable for a short period of time, and III) compounds containing more than one labile hydrogen can form multiple TMS derivatives. Owing to these drawbacks, MTBSTFA is sometimes preferred, because it produces tert-butyl dimethylsilyl (TBDMS) derivatives that are more stable and less sensitive to water [37]. Although TBDMS derivatives are more stable, the formation of TBDMS silylation requires a longer incubation time than TMS silylation. Moreover, owing to steric hindrance, the bulky TBDMS moiety can cause the incomplete derivatization of large molecules such as sugars [38].

Most studies on gut microbial AAA metabolites used TMS to derivatize the analytes [27], [28], [30]. Pautova et al. reported that the TMS derivatization of indolic acids yielded slightly better and more consistent recoveries than TBDMS derivatization [9]. As a result, TMS was chosen because of its high reproducibility and good recovery of most indolic acids in the model solution. However, TMS derivatization of a few compounds yielded low recoveries (≤ 50%) in sample matrices: 5-hydroxyindole-3-acetic acid in pooled cerebrospinal fluid and serum, indole-3-propionic acid and indole-3-lactic-acid in serum. Another derivatization method involves alkyl chloroformate using reagents such as methyl chloroformate and ethyl chloroformate [39]. With regard to AAA, Zhao et al. preferred alkyl chloroformate derivatization because of its faster and milder reaction, producing more stable derivatives within six days [26].

For absolute quantification, the matrix effect must be evaluated to ensure that the measured metabolite concentrations represent actual concentrations. The matrix effect can be minimized using several approaches, including sample dilution [40], standard addition, and matrix-matched calibration [41], and compensated using isotope-labeled internal standards [42]. Pautova et al. evaluated the matrix effect on eight targeted phenylcarboxylic acids and reported a matrix effect ranging between 5% and 30% in serum; therefore, matrix-matched calibration solutions were prepared in the serum to account for the matrix effect [27].

Because GC-MS analysis typically uses electron ionization, a hard ionization technique in which the ionization efficiency is less likely to be affected by the presence of co-eluting molecules [43], the matrix effect evaluation is sometimes overlooked when validating GC-MS methods. Although sample preparation for GC analysis can inherently reduce the matrix effect by sample dilution or can be accounted for by the addition of isotope-labeled internal standards, the inclusion of matrix effect evaluation in GC-MS development should be encouraged to ascertain the accuracy of the quantitative method.

#### 2.1.2 GC-MS and analytical performance

The chromatographic separation of AAA metabolites often relies on reverse-phase chromatography using a capillary column coated with a polysiloxane backbone modified with methyl and 5% phenylarylene as the stationary phase. A single quadrupole (SQ) MS instrument appears to be adequate for the quantitative analysis of gut microbial AAA metabolites in biological matrices, with a lower limit of quantification (LLOQ) of 400–500 nM for the measurement of phenylcarboxylic acids and indole metabolites in serum and cerebrospinal fluid (Table 2) [9], [27]. Other low-resolution MS techniques, including ion trap (IT) and time-of-flight (TOF), have also been employed to quantify a comprehensive set of host gut microbiota metabolites, including AAA metabolites [26], [31]. To assess the sensitivity of the methods, we compared the LLOQ values of metabolites measured in at least three studies. The SQ method outperformed the IT and TOF methods (Table 2). Overall, the dynamic range of the GC-MS methods ranged from one to two orders of magnitude but reached three orders of magnitude for some compounds using the TOF method (Table 2).

**Table 2:** Comparison of lower limit of quantitation (LLOQ) values (nM) of selected gut microbial AAA metabolites analyzed in more than two studies. Abbreviations: IT, Ion-trap; TOF, Time-of-flight; Q, quadrupole; SQ, single quadrupole; TRAP, orbitrap; QTRAP, quadrupole and orbitrap.

	GC-MS (IT) [30]	GC-MS (TOF) [26]	GC-MS (SQ) [27]	GC-MS (TOF) [31]	GC-MS (SQ) [9]	LC-MS/MS (TQ) [47]	LC-MS/MS (TQ) [11]	LC-MS/MS (Q-TRAP) [51]	LC-MS/MS (TQ) [58]	LC-MS/MS (TQ) [59]	LC-MS/MS (TQ) [48]	LC-MS (TRAP) [30]	LC-MS/MS (TQ) [46]
Standard solution matrix	Urine	Solvent	Serum	Feces	Solvent, cerebrospinal fluid, serum	Plasma	Solvent	Solvent	Solvent	Solvent	Solvent	Urine	Solvent
Hippuric acid	58,036	12,837		125			2260					140	
p-hydroxybenzoic acid	2919	3620	500							14		182	38
p-hydroxyphenylacetic acid	5980	3286	500	1250			284			205		1324	25
Indole-3-acetic acid		65,074		625	400	56	123	1		22	10		
Indole-3-propionic acid		119,970		438	400	3	285			41			
Phenylacetic acid	19,833	3672					397			57		5924	
Phenylpropionic acid	9987		500				719			416		1006	
5-hydroxyindole acetic acid						20		11		10			
Indoxyl sulfate						366		8	240				

Recently, two-dimensional GC-MS (GC × GC-MS) has been shown to significantly increase the peak capacity, number of metabolite detections, and metabolite identification accuracy [44]; however, compared to conventional GC-MS, it has not been widely used to quantify AAA metabolites.

### 2.2 LC-MS

Untargeted analysis of gut microbial metabolites often employs LC-MS [10], [17], [45], which provides better metabolite coverage than GC-MS or capillary electrophoresis (CE)-MS, particularly for semi-volatile and non-volatile metabolites. Nonetheless, LC-MS can be biased by sample preparation, column selection, or ionization modes because no single condition can provide a complete picture of the metabolome of a given sample. For metabolite separation, a nonpolar column is often used to screen AAA metabolites [10], [17], [45]. Wang et al. used two non-polar columns with different bonded phases, a C8 column for positive (ESI^+^) and a C18 column for negative (ESI^-^) ionization modes. The study yielded 6600 features (unidentified metabolites) in serum samples, of which 180 were identified using an in-house database [10]. Consequently, four gut microbial metabolites of AAA, including *p*-cresol, indoxyl sulfate, hippuric acid, and phenylacetylglycine, were quantified using another reversed phase, pentafluorophenylpropyl, which provides different retention and selectivity compared to the C18 and phenyl columns.

#### 2.2.1 Sample preparation

##### 2.2.1.1 Serum/plasma

Starting volumes of serum/plasma and urine range between 20 and 100 µL and 10–150 µL, respectively. Such variations in sample volume depend on the existing concentrations of targeted gut microbial AAA in the biological fluids. Similar to the sample preparation for GC-MS analysis, preparing serum/plasma and urine for LC-MS analysis begins with protein precipitation using MeOH, followed by acetonitrile (ACN) or a mixture of ACN and isopropanol [46]. Anesi et al. precipitated proteins and removed phospholipids from human plasma using commercial Ostro 96-well plates and ACN containing 1% formic acid [47]. Dodd et al. used 6% aqueous sulfosalicylic acid to precipitate proteins from serum samples, followed by incubation at room temperature for 5 min [17].

##### 2.2.1.2 Urine

Lyophilization appears unnecessary for urine samples; only one study used freeze-dried urine samples before extracting the urine residue with MeOH [48]. Simple extraction methods involving vortexing and centrifugation are commonly used after protein precipitation. After extraction, the supernatant was either dried and reconstituted in solvents or directly analyzed using LC-MS. Ordonez et al. developed an LC-MS method to analyze a wide range of urinary microbial metabolites produced after orange juice consumption [30]. The study compared the recoveries of targeted metabolites derived from simple urine extraction (i.e., dilution and centrifugation) with those derived from the SPE method. With regard to gut microbial AAA metabolites, the simple extraction method provided a performance comparable to that of the SPE method, suggesting that sample purification may not be necessary.

##### 2.2.1.3 Solid samples

Solid samples such as feces, intestinal contents, and tissue samples require additional sample preparation steps to homogenize and prevent the transformation of metabolites after sample collection (i.e., microbial fermentation and oxidation). Homogenization and cold storage are recommended to reduce sampling bias and preserve the original structures and concentrations of the metabolites [49]. Lyophilization is a common pretreatment for removing or controlling the water content of solid samples, and is a crucial step in absolute quantification. Fecal extraction often uses a mixture of MeOH and water with varying mixing ratios or 50% aqueous ACN [50]. Brief vortexing and centrifugation seemed adequate for extracting AAA metabolites from the fecal matrix. Alternatively, Dodd et al. extracted cecal content with ACN using a bead beater homogenizer to disrupt microbial cells and tissues before centrifugation [17]. For tissue samples, sample pretreatment includes snap freezing or lyophilization, homogenization, followed by centrifugation [51], [52]. Pure MeOH, or a mixture of MeOH and water, is commonly used to extract AAA metabolites from tissue samples.

##### 2.2.1.4 Bacterial cultures

Sample preparation for the bacterial culture supernatant was similar to that for the biological fluids. Dodd et al. precipitated proteins from a bacterial culture supernatant using MeOH, which was then incubated at room temperature, followed by centrifugation, evaporation of the supernatant to dryness, and reconstitution in organic solvents and 0.5% BSA [17].

Many LC-MS methods do not require derivatization for analyzing gut microbial AAA, thereby making them suitable for high-throughput analysis. However, a quantitative analysis of Tyr host microbial metabolites in plasma and urine developed by Letertre et al. adopted derivatization using a mixture of ammonium hydroxide and dansyl chloride solutions [46]. Dansylation is commonly used for the fluorescent detection of AA and the conversion of phenols and amines to dansylated derivatives that are easily ionizable by LC-MS [53]. Although dansylation helps improve the detection of phenols, the stability of the derivatized samples is a concern when stored for longer than one day [46]. Moreover, dansyl chloride can react violently with water, releasing toxic fumes of hydrogen chloride [54]. These issues must be considered when using this method.

#### 2.2.2 LC Column

Reverse-phase chromatography based on a C18 column [55] is often employed to analyze a wide range of metabolites, including AAA. A phenyl–hexyl column, which contains silica particles bonded to a phenyl-hexyl group, is another frequently used column. The presence of the phenyl group promotes pi-pi interactions with analytes containing an aromatic moiety, providing different retention selectivity for aromatic compounds compared with the C18 column [56]. Indeed, the phenyl-hexyl column was proven to provide better chromatographic separation and optimal retention for most of the target metabolites, especially Trp metabolites, when compared to the C18 and amide columns [11]. Hydrophilic interaction liquid chromatography (HILIC) is also used to separate polar metabolites that may not be well-resolved on a C18 column [57].

The mobile phase for reversed-phase LC generally consists of water and an organic eluent, usually MeOH or ACN. For the analysis of gut microbial AAA metabolites, a combination of organic solvents (MeOH, formic acid in MeOH, or formic acid in ACN) and basic or acidic aqueous solutions is commonly used. Although ACN generates a lower back pressure than MeOH, it provides a higher elution strength than MeOH, which may not resolve highly polar compounds such as indole derivatives [58]. In the same study, nine AAA metabolites were eluted during a stable 30:70 (v/v) water: MeOH gradient with the total run time of 12 min [59]. On the other hand, Tyr and Trp metabolites were eluted at lower mixing ratio of water: ACN, ∼ 25–50% [46], [48]. Zeng et al. evaluated the chromatographic performance of different aqueous eluents containing formic acid, ammonium formate, or both at different mixing ratios [59]. They reported that 0.05% formic acid yielded better peak shapes and optimal retention times for the analysis of nine microbially derived AAA metabolites (Table 1) in the ESI^-^ mode.

#### 2.2.3 Mass spectrometry

LC-MS/MS, particularly triple quadrupole (TQ) MS, has become the standard platform for quantitative analysis owing to its excellent sensitivity, selectivity, and wide dynamic range. Most LC-MS quantitative methods have been developed using TQ in the multiple reaction monitoring (MRM) mode (MRM-TQ) (Table 1). Dodd et al. used LC-Q-TOF for global analysis and LC-MRM-TQ in the ESI^-^ for quantification [17]. To date, only one study has performed MRM analysis using LC coupled with high-resolution tandem MS, quadrupole and orbitrap (Q-OT), to quantify Trp and its microbial indole derivatives [51].

To increase metabolite coverage, a combination of ESI^+^ and ESI^-^ analyses is frequently used to profile gut microbial metabolites. Typically, the ESI^-^ analysis mode is preferred for Tyr metabolites, but ESI^+^ was only used by Letertre et al. who performed dansyl chloride derivatization [46]. The study reported an enhanced sensitivity for most phenolic compounds except for *p*-hydroxyphenylpyruvic acid and *p*-cresol sulfate. As a result, *p*-hydroxyphenylpyruvic acid was not quantified, and *p*-cresol sulfate was analyzed in ESI^-^ instead [46]. Most of Trp metabolites are analyzed in ESI^+^, but ESI^-^ is also applicable for indole-3-acetic acid, indolepropionic acid, 5-hydroxyindoleacetic acid, indoxyl sulfate, indolepyruvic acid, indoleacetic acid, indoleacrylic acid, and indolepropionic acid [17], [45], [50], [58], [59]. Phe metabolites appeared to be favorably ionized in ESI^-^. A study by Zeng et al. (2019) targeting nine gut microbial AAA metabolites in serum samples (Table 1) reported higher intensities and stabilities of their deprotonated ions [59].

The structures of AAA gut microbial metabolites that contain a carboxylic acid group allow them to readily ionize in negative ion mode forming deprotonated species. For the molecules that can ionize in both ESI modes, mode selection should take into consideration the quality of quantitative signals, e.g., chromatographic peak shape and signal-to-noise ratio, as well as other target analytes needed to be quantified in the same run.

#### 2.2.4 Analytical method performance

The convenience of sample pretreatment associated with the LC-MS methodology comes at the cost of sensitivity and column lifetime because of the overload of highly complex matrices. Because ESI is a competitive process, target analytes can suffer from the presence of multiple components in the sample matrix that are co-eluted from the column. Therefore, the evaluation of the matrix effect is crucial for developing and validating LC-MS methods. The magnitude of the matrix effect was highly dependent on the types of samples and analytical protocols used for sample preparation. AAA metabolites have been shown to exhibit varying degrees of matrix effect. A comparison of the slopes derived from the neat solvent and matrix-matched calibration curves suggested that methyl indole-3-acetate would have suffered greatly from the matrix effect if the neat solvent calibration curve had been used while the other two metabolites (indole-3-acetate and methyl indole-3-propionate) exhibited minimal matrix effects [48]. Lefèvre et al. reported poor recoveries of indole derivatives in solid samples collected from mice [51]; four known microbial metabolites of Trp including indole-3-carboxaldehyde, indole-3-acetic acid, indole-3-lactic acid, and tryptophol yielded low recoveries ranging between 48% and 60% in the intestinal matrix, indicating a strong matrix effect. Their serum and urine recoveries were satisfactory (100 ± 20%), but marginal in the liver. In the same study, indoxyl sulfate, another known microbial metabolite of Trp, exhibited good recovery from all matrices (serum, urine, liver, and intestinal contents). The authors used isotope-labeled internal standards to correct for matrix effects.

In contrast, acceptable matrix effect levels have been reported for some AAA metabolites. For example, Anesi et al. observed minimal matrix effects of 80–120% for metabolites in Trp and Tyr metabolism and other gut microbial metabolites in human plasma and urine [47]. The good recoveries of 20 Phe, Tyr, and Trp metabolites from serum, urine, and fecal matrices justified the use of calibration curves derived from the neat solvent in a study by Zou et al. [11]. Two studies by Zeng et al. also reported no significant matrix effects in rat serum, urine, or feces [58], [59].

Because LOD values were not available in many studies, we compared the LLOQ values of the selected AAA measured in at least three studies to assess the sensitivity of the method (Table 2). The LLOQ can be dependent on the biological matrices in which the analytes are quantified; however, most studies reported LLOQ values derived from calibration curves prepared from solvents. The high-resolution MRM-Q-OT method showed 1–2 orders of magnitude higher sensitivity than the MRM-TQ methods. The superior performance of high-resolution MRM-Q-OT analysis is in line with the results of our recent study that compared the analytical performance of MRM-TQ and high-resolution MRM-QTOF in a fungal matrix [60]. Our study demonstrated that high-resolution MRM-QTOF is more accurate in identifying and quantifying trace levels of triterpenoids in a complex matrix. Collectively, these results highlight the superior selectivity and sensitivity of alternative platforms, such as high-resolution Q-OT and high-resolution MRM-QTOF, compared with conventional MRM-TQ.

Ordóñez et al. evaluated two quantitative GC-MS and LC-MS methods for analyzing microbial-derived phenolic acids and aromatic metabolites in human urine [30]. They reported that LC-MS provided higher coverage of microbial metabolites, especially phenolic sulfate and glucuronide metabolites, and lower LOD and LLOQ values [30]. However, LC was coupled to a high-resolution Orbitrap MS, while GC was coupled with IT-MS. The different types of MS could partly contribute to the different analytical performances.

### 2.3 Capillary electrophoresis-mass spectrometry

The CE-MS technique is suitable for polar and charged metabolites and can be used as a complementary method to LC-MS and GC-MS analyses [61]. Although CE-MS is an attractive tool for metabolomics analysis because of its ability to simultaneously analyze the cationic and anionic forms of metabolites, the applications of CE-MS in metabolomics research are still limited compared to those of NMR, LC-MS, and GC-MS [62]. A known disadvantage of CE-MS is its low sensitivity compared to GC-MS and LC-MS; however, technical developments have been made to improve instrumental sensitivity over the years [63]. Few studies have employed CE-MS to analyze gut microbial metabolites. Most of the currently used CE-MS methods were developed and validated in the early 2000 s [64], [65], [66]. For example, Soga et al. developed a CE-Q-MS method and applied it to obtain the global profiles of AA and other polar metabolites from *Bacillus subtilis* extracts [66]. The samples were extracted using a mixture of MeOH and chloroform, and the MeOH fraction was precipitated. Cationic metabolites such as AA, amines, and nucleosides were analyzed in positive mode on a fused silica capillary (50 µm i.d. × 100 cm), where formic acid was used as the electrolyte and ammonium acetate MeOH-water was used as the sheath liquid. Overall, the method detected 1692 features, with 352 metabolites identified using authentic standards. The analysis time for each run was approximately 16 h. In 2006, Soga et al. modified a previous protocol using TOF-MS. This method has been used to quantify AA and other metabolites in mouse liver tissue and serum samples [67]. Accurate mass measurement and sensitivity provided using CE-TOFMS were improved by supplementing the sheath solution with reserpine (mass recalibration standard). This allowed the identification of all the targeted metabolites (338 metabolites) with a mass tolerance of less than 10 ppm. This method exhibited good reproducibility and linearity. One of the main factors contributing to the improved analytical performance is the TOF instrument used in this study, which provides a faster scanning rate than a quadrupole. In 2017, Mishima et al. showed a good example of the use of CE-TOFMS to analyze gut microbial metabolites [68]. This study used a CE-TOFMS method originally developed by Soga et al. in 2003 [66] to quantify gut microbial metabolites in plasma, feces, and urine samples from adenine-induced renal failure and control mice under germ-free or specific pathogen-free conditions. The metabolites from plasma, urine, and fecal samples were extracted using MeOH, similar to the sample preparation for the GC-MS and LC-MS analyses. Two gut-derived microbial AAA, indoxyl sulfate and *p*-cresyl sulfate, were detected among the 183 metabolites identified in this study.

## 3 Linking metabolites to gut microbiota

Identification of the microbial origin of metabolites or associations between gut microbes and their metabolites is frequently accomplished using a multi-method approach that includes cohort studies, animal experiments, fecal microbiota transplantation, in vitro studies, bacterial cultures, and genomics. Particularly for AAA metabolites, in 2017, Dodd and colleagues used LC-MS/MS metabolite profiling in combination with bacterial culture, genetic data, and a mouse model to characterize the metabolic pathways of AAA metabolites from *Clositridium sporogenes.* The team discovered that the reductive AAA pathway in *C. sporogenes* could generate 12 AAA metabolites, nine of which are known circulating metabolites in the host serum [17]. In 2018, Koh et al. identified imidazole propionate as a microbially produced metabolite that may contribute to the development of insulin resistance. The authors performed an untargeted analysis using LC-MS/MS and identified four plasma metabolites, dopamine sulfate, glutamate, imidazole propionate, and N-acetylputrescine, that were significantly elevated in subjects with T2D compared to those in BMI-matched subjects without T2D. Cross-validation of the elevated metabolites in the mouse model showed that only imidazole propionate exhibited higher plasma concentrations in conventionalized mice than in germ-free mice. To validate these results, the authors quantified plasma imidazole propionate in a second cohort of 649 individuals. Combining the results from an in vitro experiment (gut simulator), bacterial culture, metagenomic data, animal experiments, and human cohorts, the authors concluded that imidazole propionate is a microbially produced metabolite that may contribute to the development of T2D [69]. In 2017, Saito et al. screened over hundred bacterial strains in culture media fortified with Tyr and other microbial intermediate metabolites to identify phenol-producing strains [70]. Although the validation of microbial metabolites using a mouse model and bacterial culture provided substantial evidence of microbial metabolite production, the experiment was costly and time-consuming [71]. In addition, whether the experimental results can be translated into humans remains questionable.

**Fig. 2:**
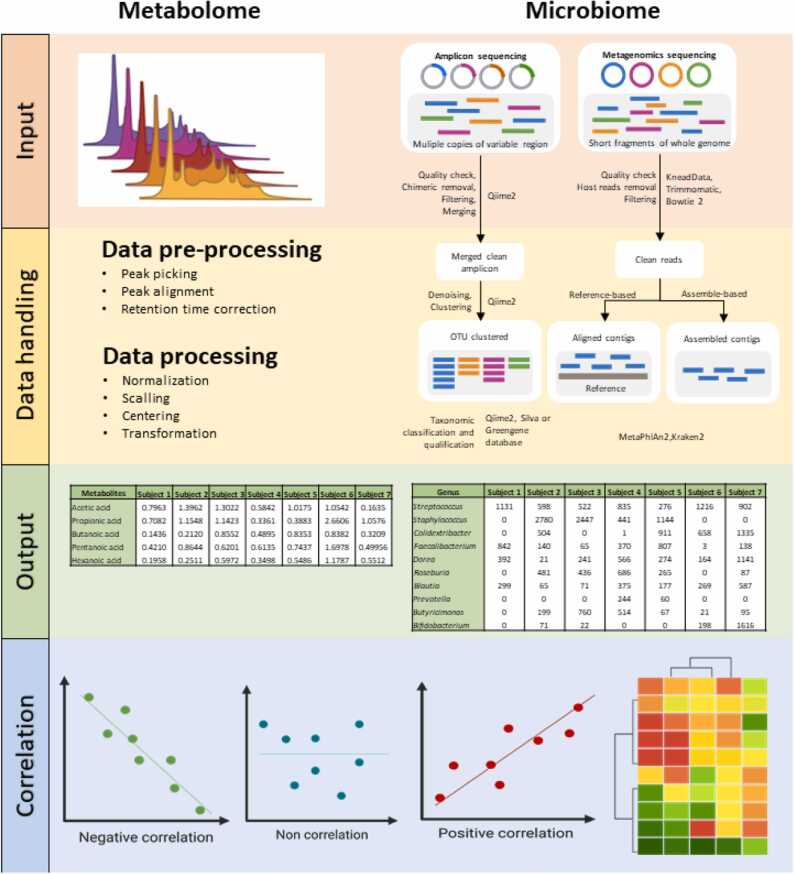
Summary workflow of data input and data handling of metabolome and microbiome before correlation analysis.

Alternatively, metabolome-microbiome integrative analysis using advanced statistical and bioinformatic tools is a crucial step in identifying gut microbes and host interplay, which can unravel the functional diversity of the gut microbiome. A summary workflow of the data input and handling processes for the metabolome and microbiome before correlation analysis is shown in Fig. 2. While metabolomic data are expressed as relative abundance (semi-quantitative analysis) or absolute concentrations (quantitative analysis) of detected metabolites, gut microbiota data can be investigated using various proxies, such as the relative abundance of Operational Taxonomic Units, read count, gut microbiome diversity, metagenomic cluster, or microbial function. One of the challenges regarding microbiome-metabolome correlation analysis is the different characteristics of the data. Microbiome data are unique due to their high dimensionality, compositionality, and sparsity, which should be considered when applying statistical analysis [72]. Microbe-metabolite correlations can be explored using classical univariate analysis such as parametric relationships (Pearson’s correlation) [73], [74], [75] and non-parametric tests (Spearman’s correlation) [10], [76], [77]. For example, Wang and colleagues characterized the gut microbiome and determined the associations between gut microbiome composition and uremic toxin metabolites (serum and fecal) in patients with end-stage renal disease and in a rat model [10]. In this study, Spearman’s correlation method was used to analyze the correlation coefficients between the functional modules of the gut microbiota and serum or fecal metabolites, whereas the random forest model was used to evaluate the correlation between the concentration of each metabolite (in serum or feces) and abundance of microbial species containing metabolite synthetase-encoding genes. Using these models, the authors identified a group of microbial species associated with uremic toxin and secondary bile acid metabolite production.

Multivariate analysis is predominantly used in the context of microbiome-metabolome investigations. Previously used multivariate analysis methods include sparse principal component analysis (PCA) [78], Co-Inertial Analysis (CIA) [79], [80], [81], Canonical correlation analysis (CCA) [78], [79], sparse CCA [82], [83], two-way orthogonal partial least squares (O2-PLS) [84], [85], and Procrustes analysis [86]. Compared to univariate analysis, multivariate analysis reduces the false discovery rate and considers the relationships between and within data matrices [86]. Liu et al. used the CIA and CCA methods to determine the associations between metagenomic linkage groups and the serum metabolome corresponding to lean and obese groups of Chinese individuals [79].

In addition to statistical correlation methods, a number of emerging bioinformatics tools enabling metabolome-microbiome or multi-omics integrative analyses have been recently developed. Incorporating multiple methods, a generalized correlation analysis for the Metabolome and Microbiome (GRaMM) was designed specifically for microbe-metabolite correlation detection. GRaMM provides data preprocessing for metabolomic and microbiome data before identifying linear and nonlinear correlations with confounder adjustment and *p*-value correction [87]. A neural network model, microbe–metabolite vectors (mmvec) deduces a microbe-metabolite interaction from the co-occurrence probability of a microbe-metabolite pair [88]. Another neural network-based model, the Microbiome-Metabolome Network (MiMeNet), uses paired microbiome and metabolomic datasets to train the model, which is then used to predict the comprehensive metabolomic profile of a given microbiome sample [89]. These newly developed tools are independent of the existing knowledge regarding microbial and host metabolic pathways, making them suitable for exploratory studies. Another approach is to exploit existing knowledge of microbial and host metabolic pathways to predict metabolic profiles based solely on microbiome sequencing data, which is beyond the scope of this review. Tools for predicting the metabolite profiles from microbiome sequencing data were evaluated and discussed [90].

You et al. evaluated six widely used correlation detection methods for integrative analysis of the metabolome microbiome: Pearson’s correlation, Spearman’s correlation, Sparse Correlations for Compositional data (SparCC), correlation inference for compositional data through Lasso (CCLasso), Mutual Information Coefficient (MIC), and cosine similarity methods [91]. They demonstrated better performance using Spearman’s correlation and MIC methods. The latter is an information-based non-parametric correlation method that measures linear and non-linear associations between variables in large datasets [92] and is also embedded in GRaMM.

## 4 Future perspectives

Owing to growing interest in the human gut microbiome and its functions, gut microbial AAA metabolites have been increasingly investigated. MS is a powerful tool not only for identifying gut microbial metabolites, but also for quantitatively analyzing complex biological samples. Over the past decade, one of the major advancements in metabolomics analysis using MS has been the addition of ion mobility spectrometry (IMS) to MS platforms. This combination has been proven to enhance the accuracy of compound identification using a rotationally averaged collision cross-section (CCS), a molecular descriptor that is derived from the time the gaseous ion travels through the drift tube and is independent of the matrices. IMS can potentially assist in novel gut microbial metabolite discovery, especially with the introduction of high-resolution cyclic IMS to enhance the separation capacity [93]. The development of CCS databases and prediction models has supported the incorporation of IMS into conventional MS-based metabolomic workflows. Current CCS databases are associated with a wide range of compound classes, but not with gut microbial metabolites. Therefore, the development of a CCS database of known and unknown gut microbial metabolites can be beneficial for large-scale profiling of metabolites and aid in novel metabolite identification. However, the use of IMS in quantitative metabolomics is limited. This is mainly due to the robustness and convenience of conventional techniques, such as LC-TQ.

While novel gut microbial metabolite discovery often exploits the power of cutting-edge mass spectrometry technology and advanced data analysis tools, quantitative analysis of known AAA microbial metabolites can be accomplished using simpler but robust measurement platforms, either LC or GC coupled with a low-resolution MS, such as TQ, Q, or TOF, which provides excellent sensitivity. In particular to the known AAA microbial metabolites, thoroughly validated quantitative methods for various biological matrices are available for both GC-MS and LC-MS platforms. But neither inter-laboratory comparison nor certified reference material has been completed for these metabolites. This concern will need to be addressed to support the implementation of these metabolites in clinical setting as accumulated evidence has demonstrated significant connections of these metabolites to multiple disease pathogenesis. Without certified reference materials, the best effort to evaluate the accuracy of a developed method is to determine recoveries of target analytes spiked in a biological matrix. Such recoveries represent the efficiency of the analytical method in recovering the free form of the analytes from the particular matrix. This may not be suitable for determining recoveries of matrix-bound analytes because the spike-in experiment may not completely mimic the actual interactions between analytes and matrix. The development of stool and urine reference materials has been initiated by the National Institute of Standards and Technology [94]. Once available, the adoption of reference materials should be encouraged, in addition to the general validation tests, to ensure the quality and accuracy of quantitative data.

## CRediT authorship contribution statement

NJ: Conceptualization, write the original draft, edit the manuscript. SK: Conceptualization, write the original draft, edit the manuscript.

## Declaration of Competing Interest’

None.
